# A Case of Myeloperoxidase Antineutrophil Cytoplasmic Antibody (MPO-ANCA)-Positive Membranoproliferative Glomerulonephritis With Latent Tuberculosis Infection

**DOI:** 10.7759/cureus.72063

**Published:** 2024-10-21

**Authors:** Ami Murakami, Hidemasa Gotoda, Takahiro Nakamoto, Tatsumasa Matsuki, Yuta Saito, Takaaki Morikawa, Shinji Lee, Akira Mima

**Affiliations:** 1 Nephrology, Osaka Medical and Pharmaceutical University, Takatsuki, JPN

**Keywords:** hematuria, latent tuberculosis infection, membranoproliferative glomerulonephritis (mpgn), mpo-anca, proteinuria

## Abstract

Membranous proliferative glomerulonephritis (MPGN), also known as mesangiocapillary glomerulonephritis, is a relatively rare glomerulonephritis with characteristic pathology. We report the case of a 77-year-old man who presented with mild proteinuria and hematuria. Laboratory tests revealed increases in myeloperoxidase (MPO)-antineutrophil cytoplasmic antibody (ANCA) titers (15.9 U/mL), negative reaction for antinuclear antibodies, hematuria, and proteinuria (3.33 g/day). The patient was a carrier of *Mycobacterium tuberculosis* with positive results in the enzyme-linked immunosorbent assay, but negative in the sputum examination. Renal biopsy revealed double contours of the glomerular basement membrane, granular deposits of immunoglobulin (Ig) G and C3 along the capillary wall, mesangial areas, and high electron density deposits in the endothelium and subepithelium, leading to the diagnosis of MPGN type 3. The patient achieved remission only with sodium-glucose cotransporter-2 (SGLT2) inhibitor without immunosuppressive drugs. Secondary MPGN can be associated with various diseases, but the relationship between elevated MPO-ANCA levels and latent tuberculosis infection remains unclear. Consequently, there have been few reports of MPO-ANCA-positive MPGN in the context of latent tuberculosis infection. Our case report suggests a possible pattern of MPO-ANCA-positive MPGN linked to latent tuberculosis.

## Introduction

Membranoproliferative glomerulonephritis (MPGN) is classified as a form of diffuse glomerulonephritis and is diagnosed by renal biopsy. It typically manifests during childhood, but it could happen at any age [1,2]. The clinical course of MPGN varies widely, with some cases presenting with rapidly progressive glomerulonephritis and others with a chronic course. Urinary findings may include hematuria and proteinuria, but severe cases of proteinuria may present with nephrotic syndrome [3,4].

Secondary MPGN can arise from various diseases, but its relationship with high myeloperoxidase (MPO)-antineutrophil cytoplasmic antibody (ANCA) and latent tuberculosis infection remains unclear. Additionally, there have been no reported cases of spontaneous remission without the use of immunosuppressive agents. In this report, we describe a MPO-ANCA-positive MPGN patient with latent tuberculosis infection who experienced spontaneous remission without the administration of immunosuppressive therapy.

## Case presentation

A 77-year-old Japanese man was referred to our hospital with mild proteinuria and microscopic hematuria, and blood sputum. He had medical histories of hypertension and aortic aneurysm, but his family history was unremarkable for any related conditions. The physical examination revealed a soft, non-tender abdomen, with no palpable spleen, liver, or kidneys. 

Upon admission, he was afebrile and his blood pressure was 134/82 mmHg. Table 1 shows the clinical data on admission. Urinalysis indicated mild proteinuria of 1.14 g/g creatinine (urine protein-to-creatinine ratio). There was no evidence of elevated immunoglobulin (Ig) or decreased complement. MPO-ANCA antibody was increased (15.9 U/mL), but there was no evidence of increases in proteinase 3-specific (PR3)-ANCA and anti-glomerular basement membrane (anti-GBM) antibody. Autoantibodies, such as anti-double stranded DNA antibodies and anti-Sm antibodies were negative. The patient was a carrier of *Mycobacterium tuberculosis*, as indicated by a positive enzyme-linked immunosorbent assay, despite a negative sputum examination.

**Table 1:** Clinical data on admission IgG: immunoglobulin G; IgA: immunoglobulin A; IgM: immunoglobulin M; C3: complement C3; C4: complement C4; MPO-ANCA: myeloperoxidase-anti-neutrophil cytoplasmic antibodies; PR3-ANCA: proteinase-3-anti-neutrophil cytoplasmic antibodies; Anti-ds-DNA Ab: anti-double stranded DNA antibody

Parameters	Patient's value	Reference values
White blood cells	5.42	3.1-8.4 (10^3^/mL)
Eosinophils	2737	100-500 (/mL)
Hemoglobin	15.1	13.0-16.6 (g/dL)
Platelet	155	15-35 (10^3^/mL)
C-reactive protein	0.18	<0.14 (mg/dL)
Total bilirubin	1.2	0.4-1.5 (mg/dL)
Aspartate aminotransferase	30	<30 (IU/L)
Alanine aminotransferase	28	<30 (IU/L)
Lactate dehydrogenase	199	120-220 (U/L)
Albumin	4.9	4.0-5.0 (g/dL)
Blood urea nitrogen	18	8-20 (mg/dL)
Creatinine	0.76	0.65-1.09 (mg/dL)
Sodium	144	137-147 (mEq/L)
Potassium	3.7	3.5-5.0 (mEq/L)
IgG	1688	870-1700 (mg/dL)
IgA	255	110-410 (mg/dL)
IgM	150	33-190 (mg/dL)
C3	144	80-140 (mg/dL)
C4	23	11-34 (mg/dL)
Rheumatoid factor	<3	<15 (IU/mL)
MPO-ANCA	15.9	<3.5 (IU/mL)
PR3-ANCA	<0.5	<2.0 (IU/mL)
Anti-ds-DNA Ab	＜1.2	<12.0 (IU/mL)
Urinalysis		
pH	6.0	4.8-7.5
Blood	2+	-
Protein	+	-
Urinary protein (g/g creatinine)	1.143	<0.15

**Figure 1:**
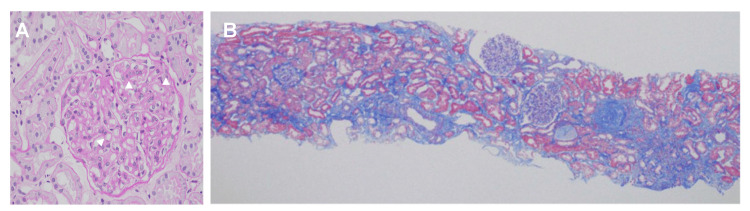
Microscopy findings of the renal biopsy (A) Double contours and segmentation in the glomerular capillaries (arrowheads) (Periodic acid-Schiff staining; Original magnification ×400) (B) Slight fibrosis in the tubulointerstitial area (Masson's trichrome staining; Original magnification ×40)

The results of the renal biopsy are as follows: Light microscopy revealed four of 24 mesangial cell proliferation, 19 of 24 mesangial expansion, and two of 24 segmental sclerosis. Double contours and segmentation in the glomerular capillaries were observed (Figure 1A). Tubular atrophy and interstitial fibrosis comprised around 20% of the total interstitial area (Figure 1B).　

**Figure 2:**
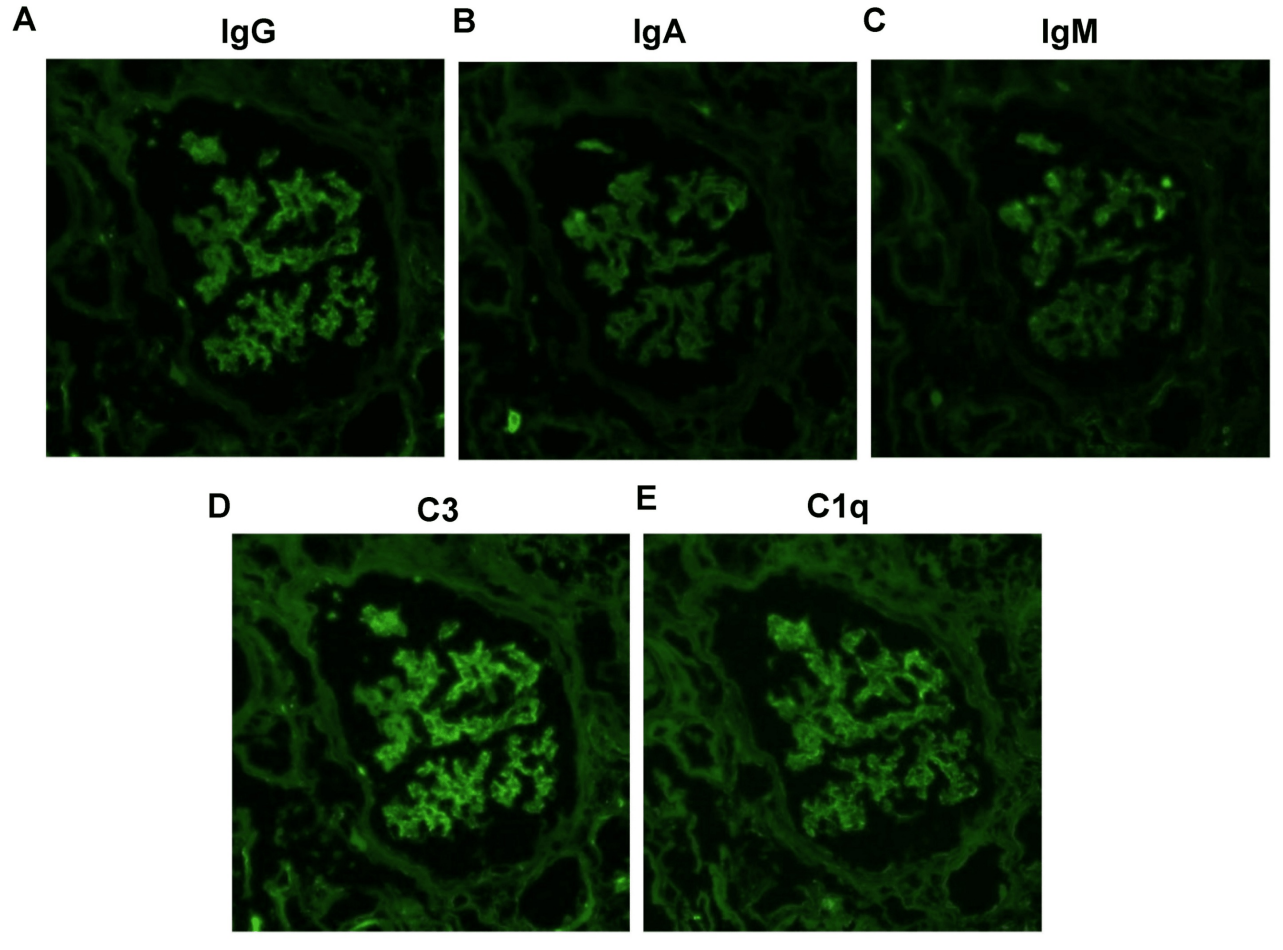
Immunofluorescence staining of the kidney biopsy (Original magnification ×400) (A) IgG (± ~1+), (B) IgA (- ~ ±), (C) IgM (- ~ ±), (D) C3 (1+), and (E) C1q (± ~1+) dominant in mesangial lesions

However, there was no pathology characteristic of microscopic polyangiitis. Immunofluorescence showed deposits of Immunoglobulin (Ig)G (± ~1+), IgA (- ~ ±), IgM (- ~ ±), C3 (1+), C1q (± ~1+) in mesangial lesions (Figure 2).

**Figure 3:**
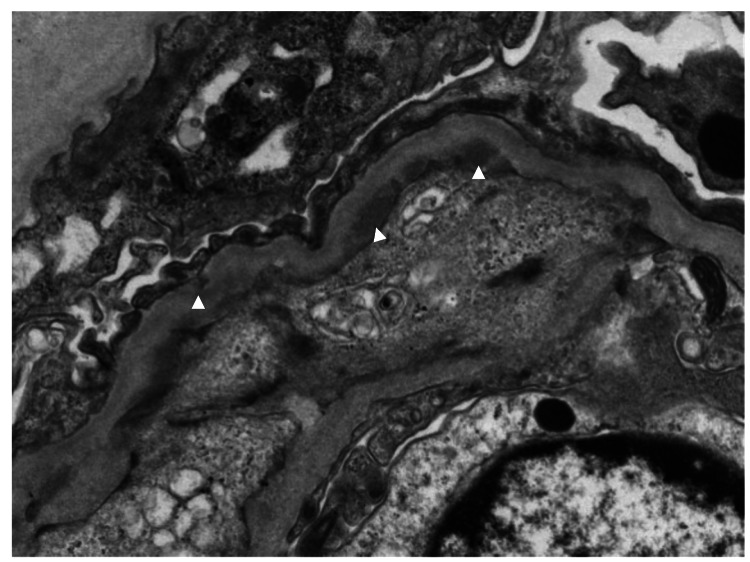
Electron microscopy showing dense deposits in the endothelium and subepithelium (arrowheads) (Original magnification ×4000)

Histopathological features were consistent with MPGN. Electron microscopy revealed moderate enlargement of epithelial cells, enlargement of the subendothelial space and mesangial invagination, and high degree of deposits in the mesangial area, subendothelium, subepithelial, and basement membrane (Figure 3).

Based on these pathological findings, a diagnosis of MPGN (type 3) was made. The patient achieved remission only with a sodium-glucose cotransporter-2 (SGLT2) inhibitor, empagliflozin (10 mg), without immunosuppressive drugs. Furthermore, during the observational period, there was no evidence of tuberculosis relapse or elevated MPO-ANCA.

## Discussion

MPGN is reported to occur almost exclusively in young adults in the age group of 8-30 years, with later onset being mostly secondary. Diseases associated with secondary MPGN are classified as follows: (a) Autoimmune diseases: systemic lupus erythematosus, Sjögren's syndrome; (b) Infectious diseases: especially hepatitis B and C; (c) Thrombotic microangiopathies: thrombotic thrombocytopenic purpura, hemolytic uremic syndrome, scleroderma; (d) Protein abnormalities: glomerular lesions due to monoclonal IgG or IgA deposition (cryoglobulinemia, multiple myeloma, many cases without amyloidosis). Clinical manifestations and progression can vary widely, spanning from benign and slowly progressive to rapidly progressive [3,5-7]. The aforementioned (a), (c), and (d) were considered unlikely in this case because of the possible relationship between latent tuberculosis and MPGN. Asymptomatic proteinuria or microscopic hematuria, acute nephritic syndrome, nephrosis, chronic kidney disease, and even rapidly progressive glomerulonephritis could be present. Blood tests usually show hypocomplementemia, with low C3, C4, and CH50 values. On the other hand, in the present case, the patient had mild proteinuria and hematuria, no hypocomplementemia, and the diagnosis was made incidentally with the results of a renal biopsy.

MPO-ANCA-associated nephritis is frequently associated with membranous nephropathy. However, the present case was considered to be MPO-ANCA-associated nephritis superimposed on type 3 MPGN. However, there have been few reports of ANCA-related CGN superimposed on type 3 MPGN, and there have been some reports of type 1 MPGN associated with MPO-ANCA [8].

It is reported that a substantial number of patients with immune complex glomerulonephritis were shown to be ANCA-positive. Furthermore, ANCA has been reported to be detected in various immune complex-mediated nephritis, post-infectious glomerulonephritis, and glomerulonephritis in heaptitis C virus (HCV) infection. Thus, immune complexes and ANCA could be associated with a subset of glomerulonephritis [9].

In patients with pulmonary tuberculosis, 2.9-25% by indirect immunofluorescence and 0-75% by enzyme-linked immunosorbent assay have been reported to be positive for MPO-ANCA [10,11]. It has been reported that humoral-mediated immunity in tuberculosis is responsible for the presence of circulating immune complexes, which can lead to glomerulonephritis; IgA antibodies, IgA immune complexes, or mycobacterial antigens targeting the A-60 mycobacterial antigen are present in the serum of patients with tuberculosis. Deposition of immune complexes, activating the lectin pathway and alternative complement pathway, with subsequent production of chemoattractant factors [12]. These factors could induce the accumulation of platelets, leukocytes, and terminal complement components (C5b-9), causing cytotoxicity [13].

Leukocytes can release proteases and oxidants which induce vascular wall damage increasing proteinuria and decreasing glomerular filtration rate [14]. Furthermore, as we have shown, inflammation and cytokines could induce glomerular damage [15-19].

A limitation of our study is that we have not been able to exclude the presence of systemic malignancy or infective endocarditis with respect to elevated MPO-ANCA.

## Conclusions

This was a case of MPGN associated with a positive MPO-ANCA, possibly due to latent tuberculosis infection. Furthermore, we were able to achieve complete remission with SGLT2 inhibitors alone, without the use of immunosuppressive drugs. Physicians should recognize that MPGN can manifest as MPGN associated with latent tuberculosis infection, even in the presence of MPO-ANCA positivity, without any signs of vasculitis.
